# Immediate Postoperative Decentration of a Biologic Corneal Inlay Managed With Tomography‐Guided Repositioning: A Case Report

**DOI:** 10.1002/ccr3.72155

**Published:** 2026-02-26

**Authors:** Giacomo Beschi, Guillermo Rocha

**Affiliations:** ^1^ Department of Medical and Surgical Specialties, Radiological Sciences, and Public Health University of Brescia Brescia Italy; ^2^ Department of Ophthalmology & Visual Sciences McGill University Montreal Canada; ^3^ Department of Surgery McGill University Montreal Canada

**Keywords:** biologic corneal inlay, corneal imaging, decentration, presbyopia, tomography‐guided repositioning

## Abstract

Biologic corneal inlays can rarely present with immediate postoperative decentration, which can be subtle and difficult to detect clinically. Real‐time tomography allows precise early diagnosis, while same‐day flap relifting and careful repositioning provide a safe, effective strategy to restore centration and preserve visual quality.

## Graphical abstract

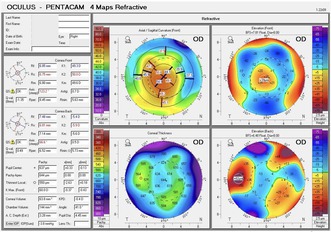

## 1 Introduction

Presbyopia affects approximately 1.8 billion people worldwide and remains a major challenge in refractive surgery [1]. Among the available surgical strategies, corneal inlays have been explored as a minimally invasive option to improve near vision while preserving distance performance.

Several synthetic corneal inlays, including the KAMRA, Raindrop, and Flexivue Microlens, were developed with different optical designs but ultimately discontinued because of biocompatibility issues and relatively high explantation rates [1]. Currently, none of these synthetic devices remain commercially available or approved in the United States.

More recently, biologic corneal inlays derived from stromal corneal tissue have been proposed as a safer and more physiologic alternative. Processed and reshaped allogeneic lenticules aim to restore near vision by modifying central corneal curvature while maintaining stromal transparency and compatibility [2, 3, 4]. Early studies by Kılıç et al. [5], Cummings [6], and Tanriverdi et al. [7] demonstrated improved near visual acuity, good refractive stability, and excellent tissue integration without reports of decentration.

In this report, we describe a case of immediate postoperative decentration of a biologic corneal inlay, a complication that, to the best of our knowledge, has not been previously documented in the literature. Prompt tomographic recognition enabled surgical repositioning and visual recovery. The case highlights the role of immediate postoperative corneal tomography as a practical strategy for early detection and precise management for maintaining optical centration and corneal integrity.

## 2 Case Presentation

### 2.1 Patient Information

A 47‐year‐old female presented seeking reduced dependence on spectacles for near vision. She was emmetropic, affected by presbyopia, and strongly motivated for surgical correction. She was otherwise healthy, with no prior ocular surgery or relevant systemic or ocular history.

**FIGURE 1:**
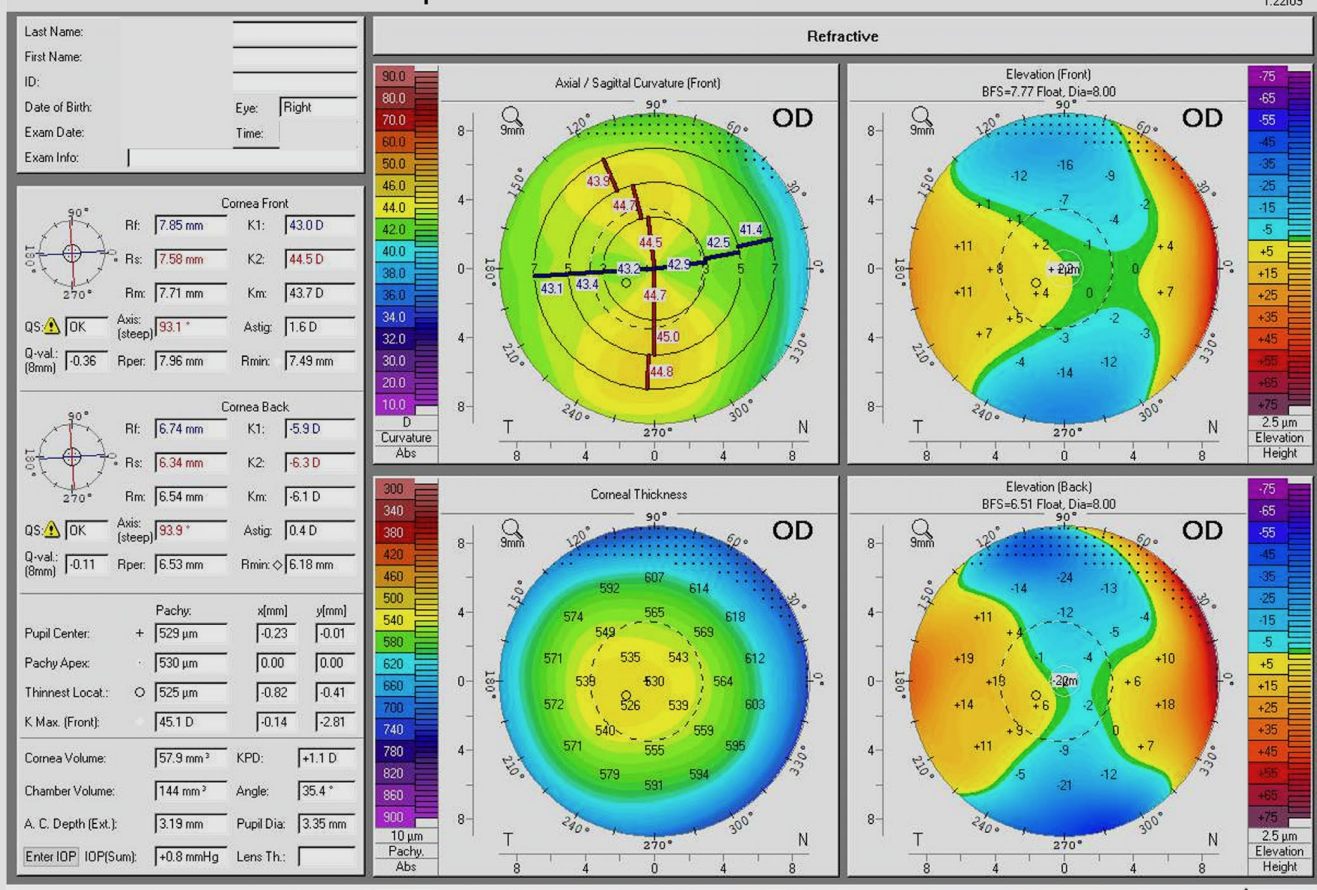
Preoperative Pentacam corneal tomography showing regular, symmetric astigmatism with normal posterior elevation and pachymetry.

The non‐dominant right eye was selected for inlay implantation. Preoperative subjective refraction was OD +0.25 sphere / +1.00 cylinder × 95° and OS +0.25 sphere / +0.25 cylinder × 40°. Corrected distance visual acuity (CDVA) was 20/15–2 letters. Central corneal thickness was 525 μm. Corneal tomography (Pentacam, Oculus Optikgeräte GmbH, Wetzlar, Germany) showed regular, symmetric anterior corneal astigmatism of 1.6 D at 93.1°, with no significant posterior ectasia (Figure 1).

### 2.2 Surgical Technique

Topical anesthesia was obtained with tetracaine 1%, followed by instillation of povidone‐iodine 5% for antisepsis. The periocular area was then gently brushed with chlorhexidine 0.05%.

An allogeneic, decellularized biologic corneal inlay (Allotex TransForm, Allotex Inc., Boston, MA, USA) was prepared according to the manufacturer's instructions. The lenticule and its glass carrier were soaked in balanced salt solution (BSS) during the preparation of the patient's corneal flap. An iFS femtosecond laser was used to create a typical LASIK flap with a thickness of 115 μm and a diameter of 8.8 mm, with a 60° hinge. Upon completion of the corneal flap, the surgical eye was prepared with a tegarderm drape and a lid speculum was placed. Linear ink marks were placed on the cornea. Under direct visualization of the microscope, the glass carrier was supported and the lenticule gently teased from the glass surface. It was then allowed to float in a metal dish, after which the dedicated Allotex loop was used to “fish out” the 22 μm inlay using capillary attraction. The patient was repositioned under the microscope, the flap was lifted and the loop/inlay was centered on the visual axis. A gentle use of a surgical spear allowed water to be removed and the inlay adhered to the corneal stroma. The flap was then repositioned and no irrigation was used in the interface. Proper alignment was ensured with the ink marks.

At the conclusion of the procedure, moxifloxacin 0.5% (Vigamox) and prednisolone acetate 1% (Pred Forte) were instilled. The surgery was completed without intraoperative complications.

### 2.3 Postoperative Evaluation and Findings

**FIGURE 2:**
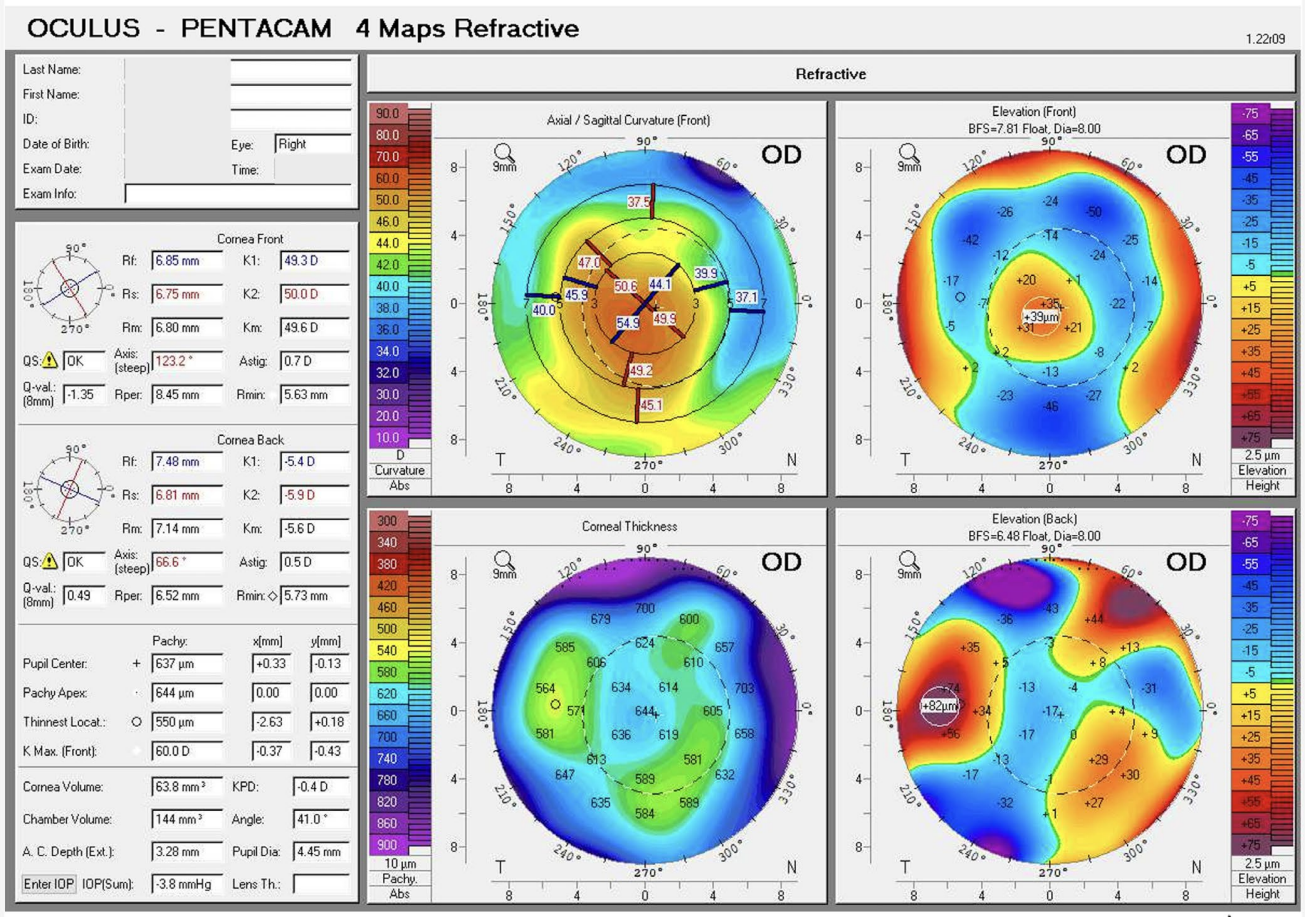
Immediate postoperative Pentacam corneal tomography revealing inferior‐temporal decentration of the biologic corneal inlay on the axial curvature map.

**FIGURE 3:**
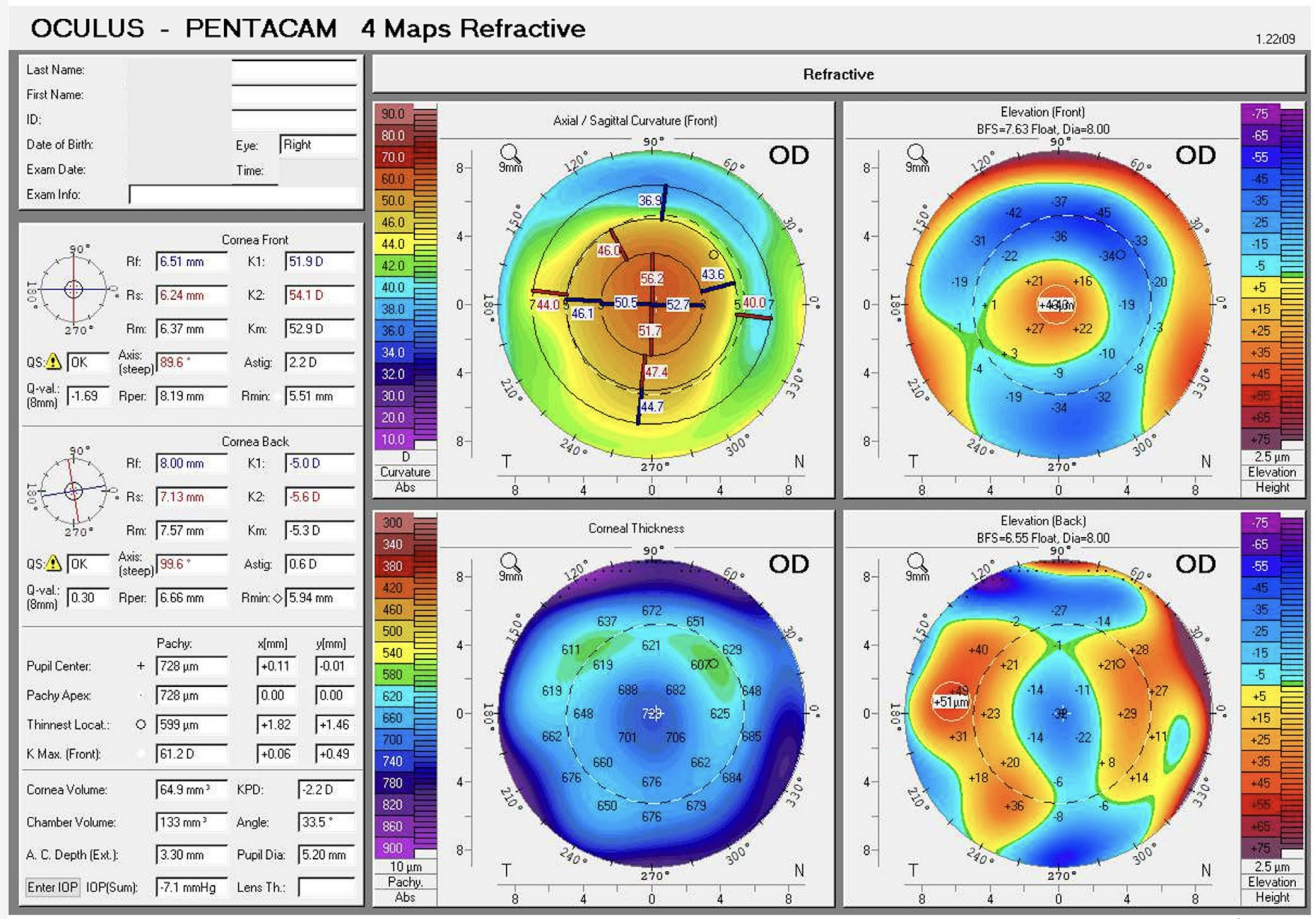
Post‐repositioning Pentacam corneal tomography confirming restoration of centration on both axial and anterior elevation maps, with uniform corneal curvature.

Immediately after the procedure, Scheimpflug tomography was repeated using the Pentacam (Figure 2). Central corneal thickness increased as expected; however, the axial map revealed slight inferior‐temporal decentration. Although an exact linear measurement of the decentration in millimeters is difficult to derive reliably from Pentacam parameters for an intrastromal inlay, the axial curvature map provided objective quantitative evidence of optical asymmetry within the central corneal zone. The 2‐mm ring showed marked asymmetry, with keratometry values of 44.1 D superonasally and 54.9 D inferotemporally. This asymmetry was even more pronounced in the 4‐mm ring (39.9 D superonasally versus 49.2 D inferiorly). The patient was therefore returned to the operating room. The flap was relifted and a small drop of BSS was placed over the inlay. A spatula was used to gently “float” the inlay and reposition it. The flap was repositioned with no interface irrigation. A subsequent Pentacam Scheimpflug tomography confirmed good centration on axial and anterior elevation maps (Figure 3). All four keratometric values provided by the Pentacam within the central 2‐mm ring were comprised between 51.7 and 56.2 D, and all four values within the 4‐mm ring ranged between 43.6 and 47.4 D, confirming restoration of symmetry within the optically relevant corneal zone.

### 2.4 Postoperative Course and Follow‐Up

On postoperative day 1, uncorrected distance visual acuity (UDVA) in OD was 20/30–2 letters, and uncorrected near visual acuity (UNVA) was J3; binocular UNVA was J2. Intraocular pressure (IOP) was 12 mmHg. Therapy included prednisolone acetate 1% q.i.d., moxifloxacin 0.5% q.i.d., and artificial tears hourly.

At 6 days, UDVA in OD was 20/40, while CDVA was 20/20–2 letters, with a manifest refraction of −2.75 D sphere / +1.25 D cylinder × 94°. IOP was 11 mmHg. The patient reported subjective improvement in near and intermediate vision but noted daily fluctuations that improved with artificial tears. This was consistent with moderate dry eye, managed with artificial tears and oral doxycycline 100 mg once daily for mild meibomian gland dysfunction (MGD).

At 6 weeks postoperatively, UDVA in the treated eye improved to 20/25–2 letters. Manifest refraction was −0.75 D sphere / +1.00 D cylinder × 85°, yielding a CDVA of 20/20–2 letters. Notably, UNVA was J2, providing excellent near visual performance. The eye was quiet, with marked improvement of MGD and dry eye. IOP was 8 mmHg.

## 3 Discussion

Previous studies on biologic corneal inlays have demonstrated encouraging outcomes in terms of visual improvement, refractive stability, and tissue compatibility, with no cases of decentration reported [5, 6, 7]. The present case adds novel evidence by documenting immediate postoperative decentration of a biologic corneal inlay and its successful management under tomography guidance. A review of the literature in PubMed and Embase did not identify previous reports of immediate postoperative decentration of biologic corneal inlays.

Decentration has been recognized as a known cause of visual disturbance in synthetic corneal inlays. Several authors have reported that even mild decentrations can lead to significant optical aberrations, glare, or reduced visual quality, often necessitating surgical repositioning of the inlay [8, 9, 10, 11]. In this regard, Sánchez‐González et al. emphasized that even a decentration as small as 0.25 mm may cause noticeable visual degradation due to the optical sensitivity of small‐aperture systems [11]. Gatinel et al. described two cases of symptomatic decentration of a small‐aperture inlay successfully managed with surgical recentering, resulting in marked visual improvement, while Hoopes et al. and Dexl et al. reported repositioning in 3%–5% of treated eyes, underscoring that centration is critical for maintaining satisfactory outcomes [ 8, 9, 10, 11].

All these reports refer to synthetic inlays, which differ substantially from biologic corneal inlays in terms of transparency, stromal integration, and biomechanical behavior. Unlike synthetic inlays, where decentration typically results from rigid body displacement of a clearly visible device, decentration of a biologic corneal inlay may occur through subtle micro‐sliding at the hydrated stromal interface, facilitated by its transparency and its biomechanical behavior, which closely resembles that of the surrounding corneal stroma.

Several technical precautions are recommended during implantation to minimize the risk of displacement, including minimal manipulation of the lenticule, controlled hydration, gentle repositioning with dedicated instruments, and avoidance of interface irrigation after flap closure. In the present case, these precautions were carefully followed. Nevertheless, due to the extremely thin, transparent, and biologic nature of the lenticule, even subtle interface fluid dynamics or minimal stromal surface irregularities may allow slight displacement to occur without being clinically apparent under the microscope. This intrinsic susceptibility to micro‐movements highlights why early tomographic assessment can be crucial, as small decentrations may not be recognized intraoperatively despite meticulous surgical technique.

In this context, the use of real‐time corneal tomography (e.g., Pentacam) immediately after implantation offers a crucial advantage. Tomography can detect subtle decentrations not apparent at the slit lamp and enables immediate correction before flap adhesion occurs. This approach may prevent delayed recognition requiring secondary surgery days or weeks later, reducing patient discomfort and minimizing the risk of interface complications. Routine intraoperative tomographic verification should therefore be considered a best practice when implanting biologic inlays. Although additional optical analysis such as corneal aberrometry could have provided further insight into the higher‐order aberrations induced by the decentration, this was not performed. Once the tomographic decentration was identified, the patient was promptly returned to the operating room, as this finding was considered clinically sufficient to justify immediate intervention. From an optical perspective, the asymmetric keratometric pattern observed on the early Pentacam maps suggests that the patient would likely have experienced marked asymmetric astigmatism within the central 2–4 mm corneal zone. Such an optical configuration is known to induce coma‐like and trefoil higher order aberrations, typically associated with glare, halos, ghosting, and reduced optical quality rather than simple loss of visual acuity. In addition, during near vision tasks, the patient might have been forced to adopt an unnatural gaze strategy in an attempt to align the visual axis with the portion of the cornea where the inlay was effectively centered. Although these effects were not formally measured, they can be reasonably inferred from the tomographic findings.

These considerations highlight the need for careful intraoperative evaluation and immediate postoperative imaging when working with ultrathin biologic corneal inlays.

## 4 Conclusion

This case illustrates how immediate postoperative tomography can identify subtle decentrations of biologic corneal inlays and enable timely repositioning before flap adhesion occurs, thereby preserving optical quality. This approach was associated with an excellent visual outcome in our patient, further supporting its clinical utility in managing early decentration of biologic corneal inlays.

Although the postoperative course in this case was uneventful, this report is limited by its single‐case nature and relatively short follow‐up, and longer observation in larger case series will be necessary to determine the incidence, risk factors, and long‐term stability of biologic inlay centration. Additional limitations include the difficulty in deriving precise linear decentration measurements from Pentacam parameters and the absence of wavefront or objective optical quality analysis. Future studies should also explore whether intraoperative imaging or customized alignment techniques could further enhance precision and reduce the likelihood of early decentration.

## Author Contributions

**Giacomo Beschi:** conceptualization, writing – original draft. **Guillermo Rocha:** conceptualization, supervision, writing – review and editing.

## Funding

This work was supported by Università degli Studi di Brescia.

## Consent

This case report did not require Institutional Review Board approval. Written informed consent was obtained from the patient for both the surgical procedure and the publication of clinical data and images, and the study was conducted in accordance with the tenets of the Declaration of Helsinki.

## Conflicts of Interest

The authors declare no conflicts of interest.

## Data Availability

Data sharing is not applicable to this article as no data sets were generated or analyzed.
